# SGLT2 Inhibitors vs. GLP-1 Agonists to Treat the Heart, the Kidneys and the Brain

**DOI:** 10.3390/jcdd10080322

**Published:** 2023-07-30

**Authors:** Bartosz Rolek, Mateusz Haber, Magdalena Gajewska, Sylwester Rogula, Arkadiusz Pietrasik, Aleksandra Gąsecka

**Affiliations:** 1st Chair and Department of Cardiology, Medical University of Warsaw, 02-097 Warsaw, Poland; bartekrolek@gmail.com (B.R.); mhaber.mateusz@gmail.com (M.H.); magdalena.gajewska@wum.edu.pl (M.G.); arkadiusz.pietrasik@wum.edu.pl (A.P.); aleksandra.gasecka@wum.edu.pl (A.G.)

**Keywords:** SGLT2 inhibitors, GLP-1-R agonists, cardiovascular outcomes, combination therapy, heart failure, chronic kidney disease

## Abstract

Sodium glucose cotransporter 2 (SGLT2) inhibitors and glucagon-like-peptide-1 receptor (GLP-1-R) agonists are novel therapeutic agents used for the management of type 2 diabetes mellitus (T2DM). Recently, large-scale randomized clinical trials have been conducted to assess the cardiovascular safety of these medications. The findings of these trials have revealed that both SGLT2 inhibitors and GLP-1-R agonists exhibit favorable cardioprotective effects, including reduction in cardiovascular and all-cause mortality, a decreased risk of chronic kidney disease progression, a decrease in hospitalization for heart failure (HF), an effect shown by SGLT2 inhibitors, and stroke prevention, an effect shown by GLP-1-R agonists. Based on the results from above studies, the European and American Diabetes Associations have issued new recommendations strongly endorsing the use of SGLT2 inhibitors and GLP-1-R agonists in combination with metformin for patients with T2DM who have additional cardiovascular (CV) comorbidities or risk factors. The primary aim of this combined therapy is to prevent CV events. Although both medication groups offer beneficial effects, they demonstrate slightly different profiles. SGLT2 inhibitors have exhibited better effects regarding a reduced incidence of HF, whereas GLP-1-R agonists have shown a reduced risk of CV events, particularly stroke. Moreover, recent European Society of Cardiology as well as American College of Cardiology and American Heart Association guidelines of HF treatment stressed the importance of SGLT2 inhibitor administration in patients with HF regardless of T2DM. In this context, we present and discuss the outcomes of the most recent trials investigating the impact of SGLT2 inhibitors and GLP-1-R agonists on renal and cardiovascular outcomes in patients, both with and without T2DM. Additionally, we explore the synergistic effects of combining SGLT2 inhibitors and GLP-1-R agonists in patients with cardiovascular disease.

## 1. Introduction

Type 2 diabetes mellitus (T2DM) constitutes one of the most widespread diseases with a prevalence of 536.6 million people in 2021 and an estimated increase to 783.2 million by 2045 [1]. It is considered to be among the major risk factors for cardiovascular disease (CVD). Patients suffering from T2DM present a two-to-four fold increased risk of developing CVD, including coronary artery disease, stroke, peripheral arterial disease, cardiomyopathy, atrial fibrillation and heart failure (HF) [2]. Furthermore, it also represents a risk factor for chronic kidney disease (CKD), which affects approximately 40% of the T2DM population. T2DM, being a cause of both macro- and microvascular damage, requires safe and efficient treatment. Particularly interesting is the mechanism in which T2DM is considered to cause CAD. In diabetic patients, particular products of myocardial metabolism, being the oxidants, may have negative impact on ion channels and thus alter their ability to modulate coronary blood flow. Such a mechanism, among others, is thought to contribute to CAD [3]. Despite the wide variety of therapeutic options available, unfortunately, the majority of glucose-lowering therapies have not shown a significant effect in lowering cardiovascular risk. In fact, a few of the medications used to lower plasma glucose level were proven to cause the opposite. For instance, notwithstanding the hypoglycemic effect of rosiglitazone, it has been shown to increase the risk of myocardial infarction (MI) by 42% and heart failure by 109% [4]. In contrast, both sodium glucose cotransporter 2 (SGLT2) inhibitors and glucagon-like-peptide-1 receptor (GLP-1-R) agonists reduce the risk of all-cause mortality, cardiovascular mortality, and kidney failure [5]. Hence, they are recommended as first-line therapy independently of background glucose-lowering agents, current human glycated hemoglobin (HbA1c) level or HbA1c targeted level in patients with T2DM and established or subclinical atherosclerotic cardiovascular disease (ASCVD) or CKD with the caveat that, in the latter, SGLT2 inhibitors ought to be preferred [6]. Although both classes appear to have a comparable effect on composite cardiovascular endpoints [7], SGLT2 inhibitors have shown a significant reduction in the risk of death or hospitalization for heart failure (hHF). Therefore, SGLT2 inhibitors were included in the latest guidelines for the diagnosis and treatment of acute and chronic heart failure of the European Society of Cardiology (ESC) [8] and presented as one of the main pillars of the recommended treatment.

In contrast, GLP-1-R agonists effectively reduce stroke events in patients with T2DM and established ASCVD, and thus have been implemented into recommendations for the stroke prevention of several associations, such as the American Stroke Association [9].

Since those two groups present different profiles of benefits, it is crucial to determine who would benefit most from which medication. 

Here, we summarized and critically interpret the available literature on (i) the mechanisms of action and the beneficial and potential adverse effects of SGLT2 inhibitors and GLP-1-R agonists; (ii) effects of SGLT2 inhibitors and GLP-1-R agonists on renal outcomes in T2DM patients; and (iii) cardiovascular benefits emerging from implying SGLT2 inhibitors and GLP-1-R agonists.

## 2. Methods

The review is based on articles found in the literature using Google Scholar, PubMed, Embase and Cochrane Library. The terms used were: “GLP-1 receptor agonists”, “SGLT2 inhibitors”, “cardiovascular”, “renal”, “benefit”, “outcome”, “empagliflozin”, “dapagliflozin”, “canagliflozin”, “sotagliflozin”, “ertugliflozin”, “dulaglutide”, “liraglutide”, “semaglutide”, “lixisenatide”, “exenatide”, “efpeglenatide”, and “albiglutide”.

This article does not contain any trials with animals or human participants conducted by the authors.

## 3. Mechanism of Action and Side Effects

### 3.1. Mechanism of Action of SGLT2 Inhibitors

SGLT2 inhibitors specifically target and block the sodium-glucose cotransporter 2 located in the epithelium of the proximal tubule of the nephron. This receptor is responsible for the reabsorption of glucose in the kidneys; thus, a blockage results in the inhibition of this process. These transporters are a suitable target for the treatment of diabetes, as they are responsible for about 90% of glucose reuptake in the kidneys. SGLT2 inhibitors show 200–2500 times greater selectivity for SGLT2 receptors compared to sodium-glucose cotransporter 1 (SGLT1) receptors, which are responsible for 10% of glucose reabsorption [10].

A notable advantage of SGLT2 inhibitors is their minimal risk of inducing hypoglycemia. This is due to compensatory mechanisms, such as increased glucose reuptake at the SGLT1 receptors and the lack of an effect on insulin secretion. Additionally, SGLT2 inhibitors promote osmotic diuresis and natriuresis, resulting in weight loss, reduced systolic and diastolic blood pressure, and lowered levels of HbA1C by approximately 0.5–1.0% [11].

The mechanisms in which SGLT2 inhibitors are considered to exert a cardioprotective effect are multiple. As one of them, the inhibition of sodium/proton exchange (NHE) is mentioned. It is thought that NHE isoform 1 is upregulated in patients suffering from HF. This isoform is of particular importance since it has been linked with cardiac hypertrophy and heart ischemia–reperfusion injury. SGLT2 inhibitors have been also found to reduce myocardial fibrosis, which constitutes one of the key factors leading to HF. Furthermore, SGLT2 inhibitors promote the utilization of ketone bodies as the energy source for the myocardium, which can lead to reduced reactive oxygen species (ROS) production. Moreover, it is thought to stabilize cell membrane integrity [12]. In terms of renoprotective effects, SGLT2 inhibitors were found to increase sodium delivery to the macula densa and thus restore the tubuloglomelural feedback. Moreover, SGLT2 inhibitors are thought to decrease GFR by constricting the afferent arteriole in the glomerulus. As mentioned before, SGLT2 inhibitors also contribute to a decrease in ROS production. A similar mechanism takes place in the glomerulus. A reduced ROS amount may lead to a decrease in the fibrosis and inflammation processes that are important factors that deteriorate renal function [13].

### 3.2. Side Effects of SGLT2 Inhibitors

The main side effects associated with SGLT2 inhibitors include polyuria caused by osmotic diuresis. Fungal infections of the genitourinary tract, affecting approximately 10% of women and 2–3% of men, have also been reported. However, there is no significant evidence suggesting an increased risk of bacterial urinary tract infections based on meta-analysis and observational studies [14,15]. Reports of skin infections, including Fournier’s gangrene, have been documented but not confirmed in large, randomized trials. Diabetic ketoacidosis, a rare side effect occurring in approximately 1% of cases, has been observed primarily in patients with type 1 diabetes. The CANVAS trial found canagliflozin to increase the risk of fractures and lower limb amputation, which was not confirmed in further trials. [11].

Overall, SGLT2 inhibitors are considered safe and well-tolerated.

### 3.3. Mechanism of Action of GLP-1-R Agonists

GLP-1-R agonists function by activating the GLP-1-R receptor in multiple organs. Glucagon-like-peptide-1 (GLP-1) is an incretin hormone produced by L-cells in the gastrointestinal tract in response to nutrient intake, particularly fat and glucose. One of the primary mechanisms of action of GLP-1 is its ability to enhance glucose-stimulated insulin secretion. Additionally, GLP-1 stimulates pancreatic islets beta-cell neogenesis, inhibits apoptosis of these cells and acts on hypothalamic receptors to promote satiety and reduce food intake. GLP-1-R agonists also slow down gastric emptying, increase glucose utilization in muscle and adipose tissues, lower blood pressure and reduce HbA1C levels. Studies have shown that liraglutide, for instance, can lead to an average decrease in HbA1C of 0.9–2.2% and a weight reduction ranging from 1.3 to 8.65 kg [11]. GLP-1-R agonists are thought to exert a cardioprotective effect by a few mechanisms, among which are the reduction in macrophage adhesion to the endothelium. This effect may lead to the reduction in atherosclerotic plaque formation [16]. Glp-1-R agonists have been also found to inhibit platelet activity ex vivo, and this mechanism can be considered as another cardioprotective factor [17].

Recent research has focused on the effect of GLP-1-R agonists on bodyweight reduction, independent of T2DM. Studies have demonstrated that semaglutide and liraglutide effectively reduce bodyweight in obese patients [18,19]. Moreover, in a recent head-to-head randomized controlled trial (RCT), semaglutide was found to be superior to liraglutide in terms of body mass reduction (difference, −9.4 percentage points; 95% confidence interval (CI), −12.0 to −6.8; *p* < 0.001) [20].

These findings provide promising evidence for the use of GLP-1-R agonists in patients struggling with obesity, potentially reducing the risk of associated complications.

### 3.4. Side Effects of the GLP-1-R Agonists

The most common side effects of GLP-1-R agonists are gastrointestinal symptoms, with nausea being reported in 25–60% of patients. However, long-acting compounds have been associated with a less frequent occurrence of nausea compared with short-acting ones. Vomiting has been observed in 5–15% of patients buy rarely leads to therapy discontinuation. Other rare side effects include injection site reactions, headaches and nasopharyngitis. GLP-1-R agonists have been associated with acute kidney injury in dehydrated patients or those experiencing severe gastrointestinal symptoms. There is no definite evidence suggesting an increased risk for pancreatitis, and meta-analyses have not shown an elevated risk of pancreatic or thyroid cancer. 

**Figure 1:**
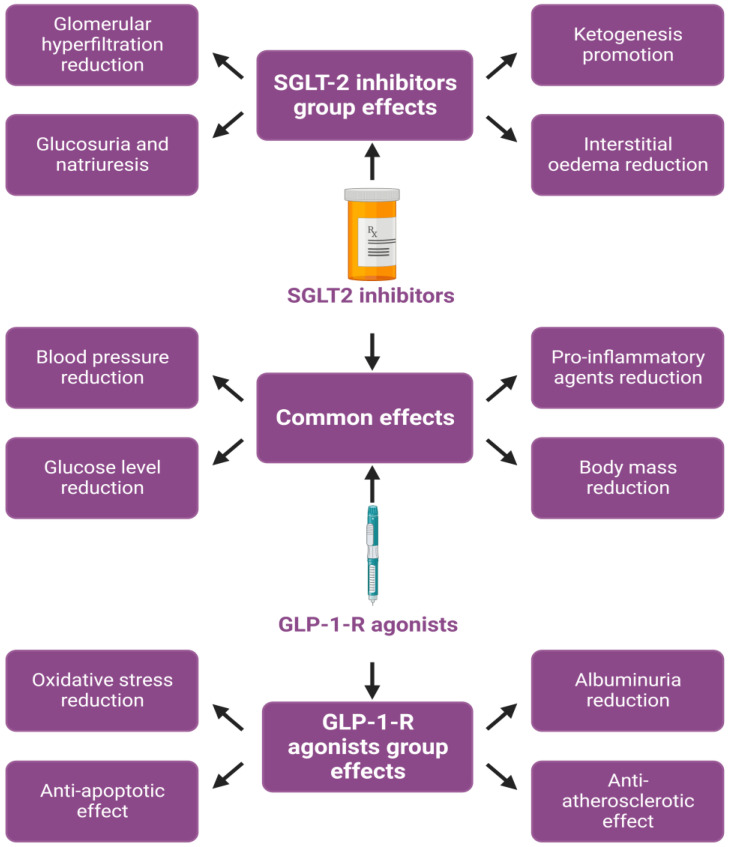
Suggested effects of SGLT2-inhibitors and GLP-1-R agonists leading to cardiorenal protection.

Therefore, GLP-1-R agonists constitute well-tolerated medications [11]. The effects of SGLT-2 inhibitors and GLP-1-R agonists that lead to cardiorenal protective benefits are presented in Figure 1.

## 4. Cardiovascular Outcomes

Both SGLT2 inhibitors and GLP-1-R agonists were found to exert a cardioprotective effect. However, the profiles of beneficial effects presented by these two groups differ. 

### 4.1. SGLT2 Inhibitors in Patients with T2DM

Several randomized controlled cardiovascular outcomes trials (CVOT) revealed the significant beneficial effects of this group of drugs on the cardiovascular system. The cardiovascular outcomes of five major RCTs in patients with T2DM are summarized in Table 1. The 3-point major adverse cardiovascular events (MACE) consist of CV death, non-fatal MI and non-fatal stroke.

The aforementioned studies were designed to evaluate the safety and efficacy of four agents from the SGLT2 inhibitors group in patients with T2DM. Those were: dapagliflozin, empagliflozin, canagliflozin and ertugliflozin. The CREDENCE trial was conducted to assess the renoprotective effect of canagliflozin in T2DM as a primary outcome.

The first published study from those mentioned above was the Empagliflozin Cardiovascular Outcome Event Trial in Type 2 Diabetes Mellitus Patients Removing Excess Glucose (EMPA-REG Outcome) trial [16], in which 7020 patients with T2DM were enrolled. They were randomly assigned to three groups: 10 mg of empagliflozin, 25 mg of empagliflozin, or placebo, all of them with their standard therapy. Subsequently, the participants were observed for a median follow up of 3.1 years. The trial found empagliflozin to reduce the risk of MACE by 14%. Moreover, it showed a significant reduction in both cardiovascular and all-cause deaths by 38% and 32%, respectively. The additional beneficial outcome was a noteworthy lower rate of hHF [21]. 

Two double-blinded, randomized trials were conducted as a part of the Canagliflozin Cardiovascular Assessment Study (CANVAS) Program [18] to evaluate the effectiveness of canagliflozin compared to a placebo in 15,494 individuals with T2DM who were at high risk of experiencing cardiovascular events. The CANVAS program recapitulated the results of EMPA-REG-outcome. Canagliflozin demonstrated significant risk reduction in MACE. However, it did not present a lower risk of cardiovascular or all-cause mortality among patients, in contrast to the EMPA-REG OUTCOME results [23].

The Canagliflozin and Renal Events in Diabetes with Established Nephropathy Clinical Evaluation (CREDENCE) trial [19] was designed to evaluate the effects of canagliflozin on renal outcomes in patients with T2DM and albuminuric chronic kidney disease. Four-thousand four-hundred-and-one individuals were recruited and administered canagliflozin 100 mg once daily or a placebo. The trial confirmed the findings of both CANVAS and EMPA-REG OUTCOME in terms of MACE [25]. The treatment with canagliflozin lowered the event rate of MACE by 20%. As in the CANVAS program, there was no significant difference between groups in terms of cardiovascular or all-cause death. Furthermore, both the CANVAS and CREDENCE trials found canagliflozin to notably reduce the risk of hHF by 33 and 39%, respectively. 

In the VERTIS trial [24], 8246 patients were enrolled and randomly assigned to ertugliflozin 5 mg, 15 mg, or a placebo once daily. The above study found ertugliflozin to reduce neither MACE nor cardiovascular or all-cause death. However, the trial showed the non-inferiority of ertugliflozin in comparison to the placebo. Nevertheless, a noteworthy reduction in hHF was shown (0.70; 95% CI, 0.54–0.90).

In the DECLARE-TIMI 58 trial, 17,160 patients with T2DM were evaluated and randomized to take either 10 mg of dapagliflozin on a daily basis or a placebo. The trial did not demonstrate dapagliflozin to be superior to the placebo in terms of MACE, cardiovascular or any-cause mortality. Nonetheless, similar to all the above studies, dapagliflozin was effective in lowering the event rate of hHF (0.73; 95% CI, 0.61–0.88) [22].

The absence of significant differences in terms of MACE events between the groups observed in DECLARE-TIMI might have resulted from the fact that patients with established CVD constituted 40% of the study population. It is plausible that a reduction in major cardiovascular events occurs only in patients with established CVD and the potential benefit of using SGLT2 inhibitors for primary prevention may not be substantial. A recent meta-analysis supports such a theorem, since it revealed that SGLT2 inhibitors reduced MACE events by 11%, but the effect was confined to patients with atherosclerotic vascular disease [26]. 

The results from the above five CVOTs were meta-analyzed by Marilly et al. It has been confirmed that the use of SGLT2 inhibitors in patients with T2DM reduced the risk of all-cause mortality and MACE by 14% (0.86; 95% CI, 0.78–0.95) and 9% (0.91; 95% CI, 0.86–0.96), respectively. Moreover, the study demonstrated a decrease in hHF risk by 31% (0.91; 95% CI, 0.86–0.96) and end-stage renal disease by 33% (0.67; 95% CI, 0.53–0.84) [27].

The trial not covered by the above meta-analysis was the Effect of Sotagliflozin on Cardiovascular and Renal Events in Patients with Type 2 Diabetes and Moderate Renal Impairment Who Are at Cardiovascular Risk (SCORED). Designed to assess the effect of sotagliflozin in patients with T2DM and CKD, it involved 10,584 patients with T2DM and eGFR of 25–60 mL/min/1.73 m^2^ of body-surface area. After a follow-up median of 16 months, sotagliflozin demonstrated a moderate reduction for the 3-point MACE by 23% (0.77; 95% CI, 0.65–0.91), whereas the risk for hHF or urgent visit for HF was reduced by 33% (0.67; 95% CI, 0.55–0.82) [28].

None of the studies discussed above presented a statistically significant beneficial effect on either MI or stroke event rate alone; therefore, SGLT2 inhibitors were not shown to be superior to the placebo in terms of stroke or MI. On the other hand, SGLT2 inhibitors have robust effects on the reduction in hHF. 

### 4.2. SGLT2 Inhibitors in Patients with HF

SGLT2 inhibitors demonstrate highly beneficial and pleiotropic effects on the cardiovascular system. Even though the exact mechanism of action remains unknown, there is an increasing tendency to evaluate their use in different CV conditions [29]. In recent years, five large RCTs, which aimed was to assess the efficacy of SGLT2 inhibitors in patients with HF, were conducted, of which four assessed patients regardless of T2DM. The main results of the aforementioned trials are presented in Table 2.

The DAPA-HF and EMPEROR-REDUCED trials were designed to assess the efficacy of dapagliflozin and empagliflozin, respectively, in subjects suffering from heart failure with reduced ejection fraction (HFrEF) [30,31]. Patients with T2DM constituted 41.8% and 49.8% of the study population, respectively. 

DAPA-HF involved 4744 patients with New York Heart Association class II, III or IV heart failure and an ejection fraction of maximum 40%. Those subjects were randomly assigned to receive either 10 mg of dapagliflozin or a placebo once daily. The follow up spanned a median of 18.2 months. Dapagliflozin demonstrated a significant 26% risk reduction (0.74; 95% CI, 0.65–0.85) in the primary outcome, which was a composite of death from cardiovascular cause or worsening of heart failure, with the latter being any unplanned hospitalization for HF or an urgent visit resulting in the intravenous administration of HF drugs. The risk reduction in the components of the primary outcome were as follows: urgent heart-failure visits markedly reduced by 57% (0.43; 95% CI, 0.20–0.90), hospitalization for heart failure by 30% (0.70; 95% CI, 0.59–0.83) and cardiovascular death rate decreased by 18% (0.82; 95% CI, 0.69–0.98). It is worth noting the impact of dapagliflozin on all-cause mortality (0.83; 95% CI, 0.71–0.97). In terms of safety outcomes, dapagliflozin was well-tolerated and did not increase the risk of volume depletion, fracture, amputation or Fournier’s gangrene.

In the EMPEROR-REDUCED trial, 3730 patients were enrolled and administered empagliflozin 10 mg or placebo once daily. During a median follow up of 16 months, empagliflozin was found to markedly lower the risk of the primary composite outcome of CV death or hHF by 25% (0.75; 95% CI, 0.65–0.86). The primary outcome was mainly related to a reduction in hospitalization for heart failure by 31% (0.69; 95% CI, 0.59- 0.81), whereas the risk ratio of CV death was non-significantly lower (0.92; 95% CI, 0.75–1.12). The beneficial effect started to be statistically significant after 12 days from randomization. Noteworthy, the risk reduction in the primary outcome was even more noticeable in patients who were receiving sacubitril-valsartan at baseline levels (0.64; 95% CI, 0.45–0.89) in comparison to subjects not taking sacubitril-valsartan (0.77; 95% CI, 0.66–0.90).

The results of DAPA-HF and EMPEROR-REDUCED were consistent within all analyzed subgroups, including patients with T2DM.

To supplement the already existing studies and reveal whether SGLT2 inhibitors exert beneficial effects in patients with heart failure regardless of ejection fraction, the EMPEROR-PRESERVED and DELIVER trials were conducted recently. Patients with T2DM represented approximately 49% and 45% of the study groups, respectively [32,33].

The EMPEROR-PRESERVED study was to evaluate the efficacy of empagliflozin in subjects with II–IV New York Heart Association class heart failure with an ejection fraction of 40% and more. A total of 5988 individuals underwent randomization and were randomly administered either empagliflozin 10 mg or a placebo once daily. As in EMPEROR-REDUCED, the key primary outcome consisted of a composite of CV death and hHF. Patients were subsequently followed for a median of 26 months. The empagliflozin arm was characterized by a 21% lower risk of primary outcome (0.79; 95% CI, 0.69–0.90), which, similar to EMPEROR-REDUCED, was largely driven by a decrease in hHF (0.71; 95% CI, 0.60–0.83). In terms of CV death, the results were also consistent with the aforementioned trial. Although the empagliflozin arm tended to have a slightly lower CV mortality rate, this effect was not statistically significant.

In the DELIVER trial, 6263 patients suffering from heart failure with an ejection fraction of more than 40% were involved. The participants were allocated at random to receive either dapagliflozin 10 mg or a placebo once daily. After a median follow up of 2.3 years, dapagliflozin demonstrated a significant 18% risk reduction in the primary outcome (0.82; 95% CI, 0.73–0.92), which consisted of CV death, unplanned visit or hHF. If analyzed separately, the risk of hHF was 33% lower in the empagliflozin arm (0.77; 95% CI, 0.67–0.89). However, the CV death and urgent visit for heart failure, while tending to a decrease in the empagliflozin group, did not reach statistical significance with HRs of (0.88; 95% CI, 0.74–1.05) and (0.76; 95% CI, 0.55–1.07), respectively. The beneficial effect of dapagliflozin did not depend on the presence of T2DM. Moreover, it did not appear to differ significantly among patients with a left ventricle ejection fraction (LVEF) of less than 60% and those with LVEF of 60% and more.

The effectiveness of the SGLT2 inhibitor sotagliflozin in worsening HF (wHF) was the subject of “The Effect of Sotagliflozin on Cardiovascular Events in Patients with Type 2 Diabetes Post Worsening Heart Failure” (SOLOIST-WHF) trial. A total of 1222 patients admitted for hHF were randomly assigned to receive either sotagliflozin or placebo. The treatment started before discharge in 48.8% or in a median of 2 days after leaving the healthcare facility in 51.2%. The follow up spanned a median of 9 months. During the trial, the primary endpoint was changed from the composite of CV death or hHF to a total number of CV death, hHF and urgent visits for HF. Such an action was taken to increase the power of the trial. In the sotagliflozin arm, the primary outcome had a 33% lower occurrence rate compared to the placebo (0.67; 95% CI, 0.52–0.85). To reduce the bias of double counting urgent visits leading to hospitalization, the total number of CV deaths and hHF was also examined. The results were consistent with those of the primary outcome (0.68; 95% CI, 0.53–0.88). In terms of CV deaths or deaths from any cause, no significant difference was demonstrated [34].

To summarize the effectiveness of SGLT2 inhibitors in patients with heart failure, Vaduganathan et al. conducted a meta-analysis of the five aforementioned trials [35]. It was demonstrated that the SGLT2 inhibitors included in the analysis (dapagliflozin, empagliflozin and sotagliflozin) markedly lowered the risk of consisted of first hHF (0.72 [0.67–0.78]), CV deaths (0.87 [0.79–0.95]), a composite of both of the above outcomes (0.74 [0.67–0.83]) and, what is more, the all-cause mortality (0.92 [0.86–0.99]) in a wide range of LVEF. Such a meta-analysis supports the use of SGLT2 inhibitors as a first-line treatment in patients with HF, regardless of LVEF or care setting. 

### 4.3. GLP-1 in Patients with T2DM 

There have been nine CVOTs regarding the use of GLP-1-R agonists in patients with T2DM.

The results of the CV outcomes of the aforementioned trials are synthesized in Table 3.

Even though all the above trials showed the non-inferiority of GLP-1-R agonists to the placebo, superiority in terms of MACE was found only in five of them:

− LEADER;
− SUSTAIN-6;
− HARMONY;
− REWIND;
− AMPLITUDE-O.

In The Liraglutide Effect and Action in Diabetes: Evaluation of Cardiovascular Outcome Results (LEADER) [36] trial, 9034 patients with T2DM were enrolled and underwent randomization in a 1:1 ratio to a group of either 1.8 mg of liraglutide or a placebo. Subsequently, the subjects were observed for a median of 3.5 years. Liraglutide vs. placebo was found to significantly reduce the risk of 3-point MACE by 13% (0.87; 95% CI, 0.78–0.97). When analyzed separately, the CV death rate was decreased by 22% (0.78; 95% CI, 0.66–0.93) in the liraglutide group, whereas non-fatal MI or non-fatal stroke did not differ between the groups [36].

The Semaglutide Unabated Sustainability in Treatment of Type 2 Diabetes 6 (SUSTAIN-6) trial [37] recruited 3297 patients with T2DM who were randomly assigned to receive once weekly semaglutide 0,5 mg/1mg subcutaneous or a placebo. Afterward, the subjects were followed for a median of 2.1 years. The trial demonstrated that semaglutide injected once weekly lowers the MACE rate by 26% (0.74; 95% CI, 0.58–0.95) with the effect largely driven by a robust reduction in the non-fatal stroke event rate (0.61; 95% CI, 0.38–0.99). The benefit of semaglutide in terms of non-fatal MI or CV death remained insignificant [37].

Semaglutide has also been an object of the PIONEER 6 trial [41]. However, the administration route differed from that of SUSTAIN-6. Once daily oral 14 mg of semaglutide was not found to decrease either the MACE rate (0.79; 95% CI, 0.57–1.11), non-fatal MI (1.18; 95% CI, 0.73–1.90) or non-fatal stroke (0.74; 95% CI, 0.35–1.57). Nevertheless, a great decrease in the number of CV deaths was demonstrated in the semaglutide arm (0.49; 95% CI, 0.27–0.92).

The recently published AMPLITUDE-O trial [40] enrolled 4076 patients with T2DM to receive either exendin-based GLP-1-R agonists, efpeglenatide, or a placebo. The follow-up time had a median of 1 year. As a result, efpeglenatide was found to significantly reduce the MACE rate by 27% (0.73; 95% CI, 0.58–0.92). Moreover, the efpeglenatide arm was characterized by the notably lower risk of hHF (0.61; 95% CI, 0.38–0.98). However, the limitation of the study was limiting the inclusion criteria to those with previous CVD or kidney disease, which lowers its generalizability in a broader population [40]. 

The HARMONY outcomes trial [38] was designed to assess the impact of albiglutide on cardiovascular outcomes. A total of 9463 subjects aged ≥ 40 with T2DM and established CVD were assigned to receive albiglutide 30 mg once weekly or a placebo. After a follow-up median of 1.5 years, the albiglutide arm had a 22% lower risk of 3-point MACE (0.78; 95% CI, 0.68–0.90). Significantly decreased was the event rate of non-fatal MI (0.75; 0.61–0.90), while both CV or all-cause mortality and stroke risk were not found to be statistically lower. 

In a recent meta-analysis, Lee et al. demonstrated that GLP-1-R agonists, despite the differences in structure and time of action, have a robust effect on the reduction in MACE (0.87; 95% CI, 0.81–0.94) and death from any cause (0.89; 95% CI, 0.83–0.95) [45]. Moreover, the previous meta-analysis performed by the above authors, which involved 60,080 patients, revealed that GLP-1-R agonists were highly effective in decreasing the relative risk of CV deaths by 13% and all-cause mortality by 12%. Noteworthily, GLP-1-R agonists also presented a significant effect on the reduction in hHF (0.89; 95% CI, 0.82 to 0.98). The possibility arises that such an effect is due to a notable reduction in MI risk and therefore to a reduced risk of HF development. Of great interest is the fact that the beneficial effect was exerted independently of the drug–dose interval. The use of agents injected once daily was associated with benefits similar to those injected once weekly [46].

Overall, the results of the aforementioned substantiated the use of GLP-1-R agonists in patients with T2DM and established ASCVD or with high cardiovascular risk. 

### 4.4. GLP-1 in Stroke Prevention

According to the results of nine CVOTs presented in Table 3, comment-worthy is also the stroke prevention effect of GLP-1-R agonists. A recent meta-analysis by Li et al. revealed that GLP-1-R agonists exert a neuroprotective effect regardless of the glycemic level. The study involved the eight CVOTs Those agents have been demonstrated to reduce the total stroke risk by 16% (0.84; 95% CI, 0.77–0.93) and non-fatal stroke by 15% (0.85; 95% CI, 0.77–0.94). However, there was no significant difference in the number of fatal strokes (0.82; 95% CI, 0.62–1.08) [47].

Although the exact molecular neuroprotective mechanisms of GLP-1-R agonists are still unknown, these agents are considered to act by decreasing the production of pro-inflammatory factors, promoting antiapoptotic mechanisms, diminishing oxidative stress and lowering advanced glycation end-products. All of the above attenuate anti-atherosclerotic effects [48,49]. Studies on animals have found that GLP-1-R agonists may increase cerebral blood flow in mice with middle cerebral artery occlusion and, therefore, improve outcomes [50]. 

Altogether, GLP-1-R agonists demonstrate a significant neuroprotective effect and, therefore, should be used as an effective and safe drug in stroke prevention strategies [49].

## 5. Renal Outcomes

### 5.1. SGLT2 Inhibitors

T2DM is associated with a higher prevalence of cardiovascular complications, and it is also a major risk factor for CKD. Several trials conducted to assess the cardiovascular outcomes of SGLT2 inhibitors and GLP-1-R agonists revealed a reduction in CKD progression in patients treated with those two groups [7].

In the EMPA-REG Outcome trial [21], in terms of renal outcomes, empagliflozin significantly reduced the risk for the composite renal endpoint (doubling of serum creatinine, progression to macroalbuminuria, initiation of renal replacement therapy or renal death). The significance of the composite renal outcome reduction was expressed the most in patients presenting with an estimated glomerular filtration rate (eGRF) greater than 90 mL/min/1.73 m^2^. However, it was still notable in individuals with an eGFR lower than 60 mL/min/1.73 m^2^ [51].

The CANVAS program found canagliflozin to reduce by 47% the composite renal defined as the sustained doubling of serum creatinine, end-stage kidney disease or death due to renal cause. Moreover, there was a slower eGFR decline and an 18% decrease in the urea–albumin–creatinine ratio in patients treated with canagliflozin than in individuals treated with a placebo. Overall, it was revealed that canagliflozin is a medication with a significant renoprotective effect. The aforementioned results were confirmed by the CREDENCE trial. The canagliflozin group had a significantly lower event rate of reaching the primary outcome consisting of end-stage kidney disease, doubling of the serum creatinine level from baseline persisting for at least one month or death from renal or cardiovascular disease [25]. 

In DECLARE-TIMI, the co-primary endpoint consisting of death from CV cause, hHF or MACE did not reach statistical significance; thus, the analyses of additional outcomes were hypothesis-generated. Hence, the renal outcomes could not be statistically assessed [22].

The DECLARE-TIMI 58, EMPA-REG and CANVAS trials were conducted to prove the non-inferiority of dapagliflozin, empagliflozin and canagliflozin, respectively, for major adverse cardiovascular events in patients with T2DM. The renal outcomes constituted a secondary endpoint. Hence, there was a need to conduct studies that considered renal outcomes as the primary endpoint.

The efficacy of dapagliflozin in patients with CKD regardless of T2DM was the subject of the DAPA-CKD trial [52], which enrolled 4304 patients with CKD and eGFR between 25 and 75 mL/min/1.73 m^2^ and a urine albumin-to-creatinine (UACR) ratio of 200 to 5000. The patients were randomly assigned to receive dapagliflozin 10 mg daily or a placebo. The primary endpoint consisted of a sustained decline in the eGFR of at least 50%, end-stage kidney disease or death from renal or CV cause. Patients were followed for a median of 2.4 years. The trial was stopped prematurely due to the efficacy of the treatment. The trial revealed that dapagliflozin decreased the risk of the primary endpoint by 39% (0.61; 95% CI, 0.51 to 0.72), with the number of patients needing treatment being nineteen.

The impact of dapagliflozin on the composite renal outcomes as well as the decline in eGFR over time was also a secondary endpoint in the DAPA-HF trial. In patients with HFrEF, it was found that dapagliflozin slowed the rate of decline in eGFR without any statistically significant effect on the composite renal outcomes [53].

EMPA-KIDNEY also supported the extension of SGLT2 inhibitors use to a wider range of patients than individuals with T2DM [54]. Six-thousand six-hundred-nine patients were randomly administered empagliflozin 10 mg once daily or the matching placebo. The patients were followed for a median of 2 years. The primary endpoint was the first occurrence of the composite outcome of kidney disease progression or cardiovascular death, which was demonstrated to be significantly lower in the empagliflozin arm. In terms of the renal outcomes alone, empagliflozin lowered the risk of kidney disease progression by 29%.

The results of large RCTs were summarized and analyzed in a recent meta-analysis [55]. In terms of the renal outcomes, it was found that SGLT2 inhibitors markedly reduce the risk of kidney disease progression by 37% and acute kidney injury by 23%. Moreover, a similar benefit was observed regardless of T2DM. 

### 5.2. GLP-1-R Agonists

GLP-1-R agonists were also shown to have a positive impact on renal outcomes.

In terms of the renal outcomes of the LEADER trial, the liraglutide group presented a 22% lower risk of nephropathy defined as the new onset of macroalbuminuria, doubling of serum creatinine level and an eGFR of ≤45 mL/min/1.73 m^2^, and renal replacement therapy and death from renal cause [12]. Although this group presented a 26% risk reduction for new-onset macroalbuminuria, a significant difference between the groups for neither the risk of doubling serum creatinine level nor renal replacement therapy was demonstrated [56].

In SUSTAIN-6, Semaglutide was found to reduce the risk for persistent macroalbuminuria, whereas no statistically significant difference was observed neither in terms of the persistent doubling of serum creatinine nor renal replacement therapy. These results are consistent with the aforementioned ones from the LEADER trial.

The Researching Cardiovascular Events with a Weekly Incretin in Diabetes (REWIND) trial [39] was a study to assess the cardiovascular safety of dulaglutide in T2DM patients compared to a placebo. The research was conducted at 371 sites in twenty-four countries and enrolled 9901 patients who were randomly administered either 1.5 mg of dulaglutide weekly or a placebo. Subsequently, the subjects were observed for a median follow up of 5.4 years. The dulaglutide group was shown to have a lower risk of composite renal outcome consisting of new-onset macroalbuminuria, a 30% decline in the eGRF or renal replacement therapy. However, it was not specified which was the major effect of composite renal outcomes on the reduction of the aforementioned.

In the AMPLITUDE-O trial, efpeglenatide markedly reduced the composite renal outcomes by 32% (0.68; 95% CI, 0.57–0.79). The above was composed of incident macroalbuminuria with an increase in UACR by at least 30%, a sustained decrease in the eGFR of at least 40% for 30 days or more and renal-replacement therapy or sustained eGFR of less than 15 mL per minute per 1.73 m^2^ for 30 days or more. The effect was mostly driven by incident macroalbuminuria with a remarkable 32% reduction, whereas the kidney function outcome event, even though numerically important, did not differ statistically between groups.

The ongoing FLOW trial [57] aims to evaluate the effect of semaglutide among patients with T2DM and CKD. There is a lack of randomized clinical trials assessing renal outcomes as primary endpoints.

## 6. Combination Therapy

The combination therapy of GLP-1-R agonists and SGLT2 inhibitors in patients with T2DM was the subject of several RCTs. The recent meta-analysis by Li et al. [58] included data polled from eight trials, which resulted in 1895 patients being analyzed.

The previously mentioned meta-analysis found a significant reduction in the level of HbA1C in combination therapy compared to monotherapy. Moreover, a low-density lipoprotein cholesterol level, body weight and systolic blood pressure were also reduced in the combination therapy arm. Notwithstanding such beneficial effects, therapy with a combination of GLP-1-R agonists and SGLT2 inhibitors resulted in a significantly increased risk for discontinuation due to adverse effects, diarrhea and vomiting. It was also noted the greater risk for hypoglycemic events. It is noteworthy that such a side effect was observed only when adding GLP-1-R agonists to the SGLT2 inhibitor monotherapy. It should be taken into consideration that such an increased risk for hypoglycemia may be due to the simultaneous use of SGLT2 inhibitors and GLP-1-R agonists with other hypoglycemic agents, such as insulins or sulphonylureas, by some of the patients [59,60]. No beneficial effect on cardiovascular outcomes was demonstrated. There were some inconsistencies with previous studies [61,62] regarding the cardiovascular event risk or triglycerides and low-density lipoprotein decrease level. However, it might have been caused by the more strict inclusion criteria and higher number of RCTs analyzed by Li et al. [58].

In terms of the impact of combination therapy on cardiovascular events, there is a lack of RCTs concerning such as primary outcomes. The existing data are inconsistent and, thus, there is a need for further studies. 

UACR changes in patients with T2DM were the primary outcome of the Dapagliflozin, Exenatide and Combination for Albuminuria reduction in Diabetes (DECADE) trial [63]. It was a randomized cross-over clinical study enrolling roughly 20 patients. Although dapagliflozin and the combination therapy were found to markedly lower the UACR value, the differences between the combination therapy and the monotherapy with dapagliflozin were not statistically significant. The small sample size was a limitation of the study as well as its open-label design and rather short follow-up period. Therefore, likewise to the cardiovascular outcomes, further studies assessing the renal benefits of combination therapy are necessary.

## 7. Position in Guidelines and Recommendations

### 7.1. T2DM Management

Recently, in terms of T2DM management, a paradigm shift has occurred. Many RCTs revealed the cardioprotective and renoprotective effects of SGLT2 inhibitors and GLP-1-R agonists. Therefore, the current T2DM treatment was altered [64]. In 2023, the American Diabetes Association released the latest “Standards of Care in Diabetes” [6], according to which adequate treatment should be chosen based on CV risk stratification. It is considered that the main goal in high CV risk patients should be the reduction in cardiorenal risk, which can be achieved by the use of either SGLT2 inhibitors or GLP-1-R agonists. The choice of the exact drug is made depending on the comorbidities. SGLT2 inhibitors are preferred to GLP-1-R agonists in patients with documented HF or CKD and should constitute the first-line treatment. Such treatment should not be only one but should be parallel to adequate primary disease medication. In subjects with ASCVD or high CV risk, the guidelines do not prioritize SGLT2 inhibitors over GLP-1-R agonists; therefore, they can be used interchangeably.

The combination therapy of SGLT2 inhibitors and GLP-1-R agonists is endorsed by the ADA. However, such a combination should be considered in patients with T2DM and ASCVD/Indicators of High Risk or CKD who have not reached the target HbA1C despite the use of SGLT2 inhibitor or GLP1-R agonist in monotherapy. Nonetheless, Diabetes Poland, in the recently released guidelines, recommends the combination therapy of SGLT2 inhibitors with GLP-1-R agonists and metformin in patients with ASVCD or multiple risk factors regardless of the target HbA1C. Moreover, these guidelines also support the choice of GLP-1-R agonists as the first-line therapy in patients with multiple ASCVD risk factors [65].

### 7.2. HF Management

In 2021, the ESC released the latest guidelines for the diagnosis and treatment of acute and chronic heart failure. A notable change was incorporated in terms of chronic heart failure management with reduced EF. Not only was the use of SGLT2 inhibitors implemented, but what is more, they were included in the class I recommendation as a first-line treatment. This modification was implemented based on the results of the DAPA-HF and EMPEROR-REDUCED trials, which have shown substantial beneficial effects on CV outcomes in patients with HFrEF [8]. Shortly after the release of the aforementioned guidelines, the results of the EMPEROR-PRESERVED trial were revealed. Recently, the DELIVER trial findings were also published. Both studies have shown a significant effect on the reduction in hHF or CV disease in patients with HFpEF or HFmrEF. Despite the outcomes of the above trials, neither empagliflozin nor dapagliflozin have been implemented by the ESC into guidelines for the management of HFpEF and HFmrEF yet.

On the contrary, in 2022, the American Heart Association (AHA) together with the American College of Cardiology in guidelines for the management of Heart Failure included the use of SGLT2 inhibitors for the treatment of both HFmrEF and HFpEF. In both situations, the use of either empagliflozin or dapagliflozin can be beneficial and is a class 2a recommendation with a moderate level of evidence derived from RCTs [66].

After the most recent ESC guidelines and findings about the usefulness of SGLT2 inhibitors in HFpEF patients, the use of SGLT2 inhibitors in patients with heart failure needs to be further investigated in order to optimize the setting of their administration [67].

It is plausible that, in the near future, the ESC guidelines are supplemented with the results of the DELIVER and EMPEROR-PRESERVED trials and that the use of SGLT2 inhibitors is recommended in patients with HF regardless of LVEF.

### 7.3. Stroke Prevention

Regarding the evidence on the neuroprotective effects of GLP-1-R agonists, they have a place in several recommendations. The 2021 AHA/American Stroke Association Guidelines for the Prevention of Stroke in Patients with Stroke and Transient Ischemic Attack support the use of GLP-1-R as an addition to metformin in patients with T2DM and established ASCVD in order to reduce stroke risk [9]. Noteworthily, the 2020 Canadian Best Stroke Practices endorse GLP-1-R agonists for individuals with T2DM who already experienced a stroke and still have not achieve the HbA1C target [68]. The introduction of GLP-1-R agonists is also substantiated by The Diabetes, Cardiorenal, and Metabolism task force, which considers such drugs to be effective in primary and secondary stroke prevention in subjects with T2DM [69]. 

Altogether, GLP-1- R agonists are considered to be effective, and the use of such agents is recommended to prevent stroke incidents in patients with T2DM and established AVSCD or at high risk of the aforementioned.

## 8. Discussion

Novel antidiabetic drugs have been proven to exert a beneficial effect on cardiorenal outcomes in patients with T2DM. However, such effect has not been demonstrated by all SGLT2 inhibitor and GLP-1-R agonist agents and, thus, physicians should be aware of within-class differences. The network meta-analysis of Wei et al. [70] has shown that, in terms of MACE risk reduction, six drugs have shown superiority compared to a placebo (empagliflozin, canagliflozin, albiglutide, liraglutide, subcutaneous (s.c.) semaglutide and dulaglutide), with s.c. semaglutide and albiglutide being most effective. Nevertheless, the above study did not include the results of AMPLITUDE-O, which found efpeglinatide to remarkably decrease the risk for MACE [40]. In the matter of hHF, canagliflozin and empagliflozin demonstrated the best efficacy, whereas dapagliflozin and empagliflozin were found to be most effective for kidney function progression.

In a meta-analysis of several CVOTs regarding SGLT2 and GLP-1-R agonists, Zelniker et al. [26] found that the beneficial effect on MACE risk reduction was restricted to patients with established ASCVD. On the contrary, a recent study by Wright et al. [71] demonstrated that SGLT2 inhibitors and the SGLT2 inhibitors/GLP-1-R agonists combination therapy may have a beneficial impact on the primary prevention of MACE. Such an inconsistency entails further RCTs for primary prevention.

To date, there are no head-to-head RCTs that compare SGLT2 inhibitors with GLP-1-R. Hence, the comparison of the above can be conducted via network meta-analyses and therefore interpreted with an awareness of their limitations. Consequently, a need for further investigations arises.

As presented, different GLP-1-R agonists and SGLT2 inhibitors exert different effects on cardiorenal outcomes. Therefore, in terms of clinical practice and treatment management, a personalized approach should be considered. In patients with T2DM and AVSCD or high-risk of CV disease, GLP-1-R agonists or SGLT2 inhibitors should be used. Regarding the 2023 guidelines by Diabetes Poland, GLP-1-R agonists may be considered superior to SGLT2 inhibitors in patients with multiple ASVCD risk factors [65]. According to several recommendations [9,68], GLP-1-Ra should also be chosen over SGLT2 inhibitors in patients at risk of stroke. Patients suffering from CKD, with eGFR > 20 mL/min per 1.73 m^2^, should be treated with SGLT2. However, GLP-1-R agonists may be considered when the aforementioned are contraindicated or not tolerated [6]. In individuals with HF, regardless of LVEF, SGLT2 should constitute a first-choice therapy. There is evidence strongly supporting the use of SGLT2 inhibitors in patients with HF or CKD independently of the presence or absence of T2DM [52,54].

Although the SGLT2 inhibitors/GLP-1-R agonists combination therapy may help in HbA1C and body weight reduction, hypoglycemia is more likely to occur compared to monotherapy [58]. Therefore, individuals treated with such a therapy should be informed of this possibility and be closely monitored by physicians. For a better evaluation of combination therapy on cardiorenal outcomes, further studies are necessary.

Altogether, SGLT2 inhibitors and GLP-1-R agonists constitute highly effective therapy agents in terms of cardiorenal outcomes. Notwithstanding those drugs being rather safe, there are some adverse effects that one should be aware of. Although originally incorporated into T2DM treatment algorithms, emerging evidence extends their use to patients without the aforementioned. The question posed in the paper’s title is not easy to answer. However, according to all data provided, both agents are important, and therefore treatment should be personalized and cover the existing comorbidities. Considering the large amount of data that is appearing at present, it is plausible that, soon, new groups of patients would benefit from the use of SGLT2 inhibitors or GLP-1-R agonists regardless of T2DM.

## Figures and Tables

**Table 1:** Summary of the major RCTs on SGLT2 in patients with T2DM.

Trial	EMPA-REG [21]	DECLARE-TIMI [22]	CANVAS PROGRAM [23]	VERTIS [24]	CREDENCE [25]
Intervention	Empagliflozin 10 mg vs. placebo	Dapagliflozin 10 mg vs. placebo	Canagliflozin 100 mg vs. 300 mg vs. placebo	Ertugliflozin 5 mg vs. 15 mg vs. placebo	Canagliflozin 100 mg vs. placebo
Size of the groups	Empagliflozin: *n* = 4687 Placebo: *n* = 2333	Dapagliflozin: *n* = 8582 Placebo: *n* = 8578	Canagliflozin: *n* = 5795 Placebo: *n* = 4347	Ertugliflozin: *n* = 5493 Placebo: *n* = 2745	Canagliflozin: *n* = 2202 Placebo: *n* = 2199
Main inclusion criteria	T2DM; Established cardiovascular disease, eGFR > 30 mL/min/1.73 m^2^	T2DM; Established ASCVD or multiple risk factors; eGFR > 60 mL/min/1.73 m^2^	T2DM; Established ASCVD or multiple risk factors; eGFR > 30 mL/min/1.73 m^2^	T2DM; ≥40 years old, established ASCVD	T2DM, ≥30 years old; albuminuric CKD (UACR > 300 to 5000 mg/g)
Follow-up median (years)	3.1	4.2	3.61	3.5	2.62
Primary endpoint (95% CI)	MACE	MACE	MACE	MACE	End-stage kidney disease, doubling of the serum creatinine level from baseline, or death from renal or cardiovascular disease.
	0.86 (0.74–0.99)	0.93 (0.84–1.03)	0.86 (0.75–0.97)	0.97 (0.85–1.11)	0.80 (0.67–0.95)
Cardiovascular death; HR (95% CI)	0.62 (0.49–0.77)	0.98 (0.82–1.17)	0.87 (0.72–1.06)	0.92 (0.77–1.11)	0.78 (0.61–1.00)
All-cause death; HR (95% CI)	0.68 (0.57–0.82)	0.93 (0.82–1.04)	0.87 (0.74–1.01)	0.93 (0.80–1.08)	0.83 (0.68–1.02)
Hospitalization for heart failure or death from cardiovascular cause; HR (95% CI)	0.66 0.55–0.79)	0.83 (0.73–0.95)	0.78 (0.67–0.91)	0.88 (0.75–1.03)	0.69 (0.57–0.83)
Hospitalization for heart failure; HR (95% CI)	0.65 (0.50–0.85)	0.73 (0.61–0.88)	0.67 (0.52–0.87)	0.70 (0.54–0.90)	0.61 (0.4–0.80)
Myocardial infarction; HR (95% CI)	0.87 (0.70–1.09)	0.89 (0.77–1.01)	0.89 (0.73–1.09)	1.04 (0.86–1.26)	-
Stroke; HR (95% CI)	1.18 (0.89–1.56)	1.01 (0.84–1.21)	0.87 (0.69–1.09)	1.06 (0.82–1.37)	-

MACE—Major adverse cardiovascular events; T2DM—Type 2 diabetes mellitus; HR—Hazard ratio; CI—Confidence interval; ASCVD—Atherosclerotic cardiovascular disease; CKD—Chronic kidney disease; UACR—Urine albumin to creatinine ratio.

**Table 2:** Summary of the major RCTs on SGLT2 in patients with HF.

Trial	DAPA-HF	EMPEROR-REDUCED	DELIVER	EMPEROR-PRESERVED	SOLOIST WHF
Intervention	Dapagliflozin 10 mg vs. placebo	Empagliflozin 10 mg vs. placebo	Dapagliflozin 10 mg vs. placebo	Empagliflozin 10 mg vs. placebo	Sotagliflozin 200–400 mg vs. placebo
Size of the groups	Dapagliflozin: *n* = 2373 Placebo: *n* = 2371	Empagliflozin: *n* = 1863 Placebo: *n* = 1867	Dapagliflozin: *n* = 3131 Placebo: *n* = 3132	Empagliflozin: *n* = 2997 Placebo: *n* = 2991	Sotagliflozin: *n* = 608 Placebo: *n* = 614
Main inclusion criteria	HFrEF	HFrEF	HfmrEF; HFpEF ≥ 40 years old	HFmrEF; HfpEF	T2DM; hHF with intravenous drug administration
Follow-up median (years)	1.52	1.33	2.3	2.18	0.75
Primary endpoint (HR 95%CI)	Composite of worsening heart failure or death from cardiovascular causes *	Death from cardiovascular causes or hHF	Composite of worsening heart failure or death from cardiovascular causes *	Death from cardiovascular causes or hHF	Death from cardiovascular causes or hHF ^
	0.74 (0.65–0.85)	0.76 (0.65–0.86)	0.82 (0.73–0.92)	0.79 (0.69–0.90)	0.67 (0.52 to 0.85)
Cardiovascular death; HR (95% CI)	0.82 (0.69–0.98)	0.92 (0.75–1.12)	0.88 (0.74–1.05)	0.91 (0.76–1.09)	0.84 (0.58 to 1.22)
All-cause death; HR (95% CI)	0.83 (0.71–0.97)	0.92 (0.77–1.10)	0.94 (0.83–1.07)	1.00 (0.87–1.15)	0.82 (0.59 to 1.14)
Hospitalization for heart failure or death from cardiovascular cause; HR (95% CI)	0.75 (0.65–0.85)	-	0.77 (0.67–0.89) **	-	0.72 (0.56 to 0.92) ***
Hospitalization for heart failure; HR (95% CI)	0.70 (0.59–0.83)	0.69 (0.59–0.81)	0.77 (0.67–0.89)	0.71 (0.69–0.9)	0.64 (0.49 to 0.83) ****

* An episode of worsening heart failure was either an unplanned hospitalization or an urgent visit resulting in intravenous therapy for heart failure. ** Total no. of worsening heart failure events and cardiovascular deaths was measured. Worsening heart failure events were defined as hospitalization for heart failure or an urgent visit for heart failure. The total number of worsening heart failure events included first and recurrent events. *** Deaths from cardiovascular causes, hospitalizations and urgent visits for heart failure, and events of heart failure during hospitalization. **** Hospitalizations and urgent visits for heart failure. ^ Changed to the total number of deaths from cardiovascular causes and hospitalizations and urgent visits for heart failure (first and subsequent) during trial. HFrEF—Heart failure with reduced ejection fraction. HFmrEF—Heart failure with mildly reduced ejection fraction. HFpEF—Heart failure with preserved ejection fraction. HR—Hazard ratio. CI—Confidence interval. T2DM—Type 2 diabetes mellitus. HHF—Hospitalization for heart failure.

**Table 3:** Summary of the major RCTs on GLP-1-R agonists in patients with T2DM.

Trial	LEADER [36]	SUSTAIN-6 [37]	HARMONY [38]	REWIND [39]	AMPLITUDE-O [40]	PIONEER 6 [41]	EXSCEL [42]	ELIXA [43]	FREEDOM [44]
Intervention ** subcutaneous (if not specified otherwise)	Liraglutide 1.8 mg vs. placebo once daily	Semaglutide 0.5 mg vs. 1 mg vs. placebo once weekly	Albiglutide 30 mg vs. placebo once weekly	Dulaglutide 1.5 mg vs. placebo once weekly	Efpeglinatide 4 mg vs. 6 mg vs. placebo once weekly	Semaglutide 14 mg vs. placebo once daily per os	Exenatide 2 mg vs. placebo once weekly	Lixisenatide 20 μg vs. placebo once daily	Exenatide vs. placebo in a continuous subcutaneous infusion
Size of the groups	Liraglutide: *n* = 4668 Placebo: *n* = 4672	Semaglutide: *n* = 1648 Placebo: *n* = 1649	Albiglutide: *n* = 4731 Placebo: *n* = 4732	Dulaglutide: *n* = 4949 Placebo: *n* = 4952	Efpeglinatide: *n* = 2717 Placebo: *n* = 1359	Semaglutide: *n* = 1591 Placebo: *n* = 1592	Exenatide: *n* = 7356 Placebo: *n* = 7396	Lixisenatide: *n* = 3034 Placebo: *n* = 3034	Exenatide: *n* = 2075 Placebo: *n* = 2081
Main inclusion criteria	T2DM; ≥50 years old with established CVD, ≥60 years old with at least one risk factor	T2DM; ≥50 years old with established CVD, heart failure or CKD stage ≥ 3; ≥60 years old with at least one risk factor	T2DM; ≥40 years old with established CVD	>50 years old, previous cardiovascular event, CVD or multiple cardiovascular risk factors	T2DM; ≥50 (male)/55 (female) with CKD with at least 1 CV risk factor; ≥18 years old with established CVD	T2DM; ≥50 years old with established CVD or CKD, ≥60 years old with at least one risk factor	T2DM; established CVD (70% of study group)	Acute coronary syndrome at 180 days prior to screening	T2DM; ≥40 years old with established CVD, ≥60 years old with at least one risk factor
Follow-up median (years)	3.8	2.1	1.5	5.4	1.81	1.33	3.2	2.08	1.33
Primary endpoint (95% CI)	3-point MACE	3-point MACE	3-point MACE	3-point MACE	3-point MACE	3-point MACE	3-point MACE	4-point MACE	4-point MACE
	0.87; 0.78 to 0.97	0.74 (0.58–0.95)	0.78 (0.68–0.90)	0.88 (0.79–0.99)	0.73 (0.58–0.92)	0.79 (0.57–1.11)	0.91 (0.83–1.00)	1.02 (0.89–1.17)	1.21 (0.90–1.63)
Cardiovascular death; HR (95% CI)	0.78 (0.66–0.93)	0.98 (0.65–1.48)	0.93 (0.73–1.19)	0.91 (0.78–1.06)	0.72 (0.50–1.03)	0.49 (0.27–0.92)	0.88 (0.76–0.97)	0.98 (0.78–1.22)	1.22 (0.70–2.12)
All-cause death; HR (95% CI)	0.85 (0.74–0.97)	1.05 (0.74–1.50)	0.95 (0.79–1.16)	0.90 (0.80–1.01)	0.78 (0.58–1.06)	0.51 (0.31–0.84)	0.86 (0.77–0.97)	0.94 (0.78–1.13)	1.20 (0.79–1.81)
Hospitalization for heart failure; HR (95% CI)	0.87 (0.73–0.97)	1.11 (0.77–1.61)	-	0.93 (0.77–1.12)	0.61 (0.38–0.98)	0.86 (0.48–1.55)	0.94 (0.78–1.13)	0.96 (0.75–1.23)	0.95 (0.48–1.88)
Myocardial infarction; HR (95% CI)	0.86 (0.73–1.00)	0.74 (0.51–1.08)	0.75 (0.61–0.90) *	0.96 (0.79–1.15)	0.75 (0.54–1.05)	1.18 (0.73–1.90) *	0.97 (0.85–1.10)	1.03 (0.87–1.22)	1.33 (0.82–2.17) *
Stroke; HR (95% CI)	0.86 (0.71–1.06)	0.61 (0.38–0.99)	0.86 (0.66–1.14)	0·76 (0·62–0·94)	0.74 (0.47–1.17)	0.74 (0.35–1.57) *	0.85 (0.70–1.03)	1.12 (0.79–1.58)	1.00 (0.56–1.79)

* Non-fatal myocardial infarction or stroke. ** Subcutaneous (if not specified otherwise). T2DM—Type 2 diabetes mellitus. MACE—Major adverse cardiovascular events. HR—Hazard ratio. CI—Confidence interval. CVD—Cardiovascular disease. CKD—Chronic kidney disease. CV—Cardiovascular.

## Data Availability

No new data were created or analyzed in this study. Data sharing is not applicable to this article.
